# Local-Regional Recurrence of Pheochromocytoma/Paraganglioma: Characteristics, Risk Factors and Outcomes

**DOI:** 10.3389/fendo.2021.762548

**Published:** 2021-10-19

**Authors:** Yunying Cui, Xiaosen Ma, Yinjie Gao, Xiaoyan Chang, Shi Chen, Lin Lu, Anli Tong

**Affiliations:** ^1^ Key Laboratory of Endocrinology, Department of Endocrinology, National Health Commission of the People’s Republic of China, Peking Union Medical College Hospital, Peking Union Medical College, Chinese Academy of Medical Sciences, Beijing, China; ^2^ Department of Pathology, Peking Union Medical College Hospital, Peking Union Medical College, Chinese Academy of Medical Sciences, Beijing, China

**Keywords:** recurrence, characteristics, risk factors, outcomes, pheochromocytoma/paraganglioma

## Abstract

**Objective:**

To study the characteristics, risk factors, and outcomes of local-regional recurrence of pheochromocytoma and paraganglioma (PPGL).

**Methods:**

Clinical data of 96 PPGL patients with local-regional recurrence and 112 patients without recurrence were retrospectively analyzed.

**Results:**

Recurrent patients exhibited a median recurrence time of 6.0 (4.0, 9.0) years after resection of the primary tumor. *SDHB* mutation [HR 4.1 (1.7, 9.5), p=0.001), primary tumor size ≥5cm [HR 2.3 (1.1, 4.7), p=0.028], and average Ki-67 count ≥3% in the primary tumor [HR 2.6 (1.4, 4.9), p=0.003] were independent predictors for recurrence of PPGL. Primary tumor sizes ≥5cm [HR 5.1 (1.7, 15.3), p=0.003] and average Ki-67 counts ≥3% of the primary tumor [HR 2.4 (1.1, 5.2), p=0.035] were independent predictors for recurrence of pheochromocytoma, while *SDHB* mutation [HR 4.6 (1.5, 13.9), p=0.007] was a predictor for paraganglioma recurrence. Among recurrent patients, 47% (45/96) had multiple nodules in recurrent sites, and 58% (56/96) had metastases, with 20% (19/96) being implantation metastases. The risk of metastases (42% vs. 25%, p=0.030) and death (15% vs. 8%, p=0.003) was significantly increased in untreated patients after recurrence compared with treated patients.

**Conclusion:**

Long-term follow-up is necessary for all PPGL patients. Risk factors for recurrence of pheochromocytoma and paraganglioma differ, with primary tumor size and average Ki-67 count representing independent predictors for pheochromocytoma patients and *SDHB* mutations predicting paraganglioma recurrence. Although the treatment of recurrence can be difficult, patients should be treated once recurrence is detected as it decreases the risk of metastases and death.

## Introduction

Pheochromocytoma (PHEO) and paraganglioma (PGL), together referred to herein as PPGL, are rare neuroendocrine tumors arising from chromaffin cells of the adrenal medulla and extra-adrenal autonomic paraganglia, respectively. The incidence of PPGL is approximately 0.6 cases per 100,000 person­years (1). Once PPGL is diagnosed, surgery is the mainstay of treatment. Long-term follow-up is recommended for patients who have undergone surgery as even tumor-free patients are at risk of recurrence and metastases. Recurrence after resection is reported to occur in 3-16% of patients (2–7) and can be difficult to treat, especially if diagnosis is delayed. So far, few studies focused on the characteristics of local-regional PPGL recurrence, all which were small scale studies (2, 3, 7). The aim of this study was to study the characteristics, risk factors, and outcomes of local-regional recurrence of PPGL. The data presented in this article represents the largest study of PPGL recurrence to date.

## Methods

### Patients

We retrospectively studied clinical data of 96 patients diagnosed with local-regional recurrent PPGL in Peking Union Medical College Hospital, China. Local-regional recurrence referred to a reappearance of disease at the original site with or without metastases after complete surgical resection of the original tumor which had been confirmed by negative biochemical and imaging tests. Radiological features of local recurrence were often irregular in shape and closely adhered to surrounding tissues, and may be multiple nodules fused together. The recurrence-free survival was defined as the time elapsed from initial surgery until recurrence. Patients with metastases at onset, failure of complete resection of the primary tumor or new lesions which were not located at the original site of disease were excluded. We also retrospectively analyzed clinical data of 112 patients without recurrence with a median follow-up time of 8.0 (7.0, 9.8) years to study risk factors for recurrence. Patients without local-regional recurrence referred to those patients without recurrence or metastasis after complete resection of the primary tumor during the entire follow-up. Metastases were defined in accordance with the 2017 WHO classification of endocrine tumors. Metastases-free survival was defined as the time elapsed from initial surgery until metastasis. Clinical data, including gender, age at diagnosis, recurrence and metastases, symptoms, blood pressure (BP), primary tumor location, the maximum diameter of primary tumor, tumor secretion function (24-hour urinary catecholamine excretion), primary tumor pathology, genetic characteristics, treatment, and prognosis of recurrence were collected. The study was approved by the Institutional Review Board of Peking Union Medical College Hospital(S-K431). Written informed consent was obtained from all included patients.

### Statistical Analysis

All statistical analyses were conducted using Statistical Product and Service Solutions, version 21.0. Categorical data are presented as frequencies and percentages. Normally distributed data are expressed as mean ± standard deviation (x̄ ± s). Non-normally distributed data are presented as median and quartile range (25%, 75%). The association between two independent samples with a normal distribution was determined using independent sample T tests, while the association between two independent samples with a non-normal distribution was assessed using Wilcoxon rank sum tests. Associations between two dichotomous parameters were evaluated utilizing the Chi-square test. Kaplan-Meier testing was employed to describe progression-free recurrence. Factors validated in univariate analysis were further tested in multivariate analysis using Cox proportional hazard models. Results are reported as hazard ratios (HR) with 95% confidence intervals. All tests were conducted two-sided, with P values< 0.05 being considered statistically significant.

## Results

### Patient Characteristics

**Figure 1:**
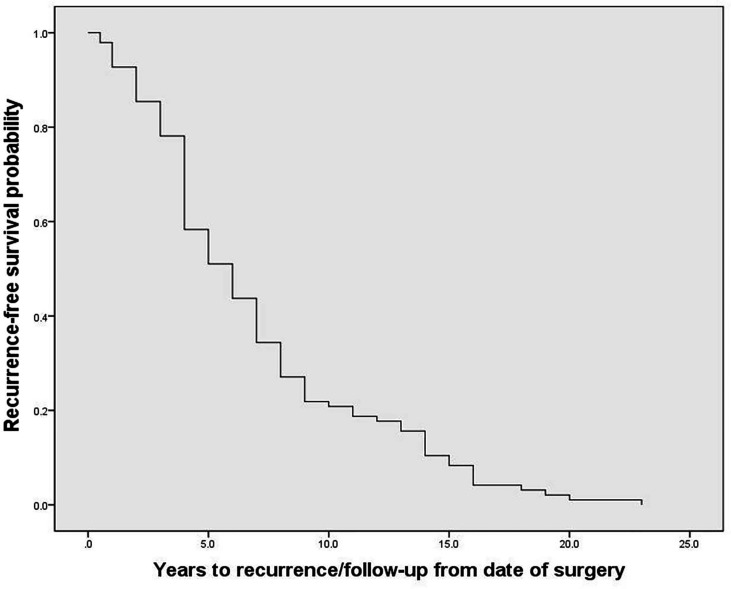
The recurrence-free survival of recurrent patients with pheochromocytoma and paraganglioma.

The 96 PPGL patients with local-regional recurrence included in this study, consisting of 67 PHEO patients and 29 PGL patients, exhibited an average diagnosis age of 32.2 ± 14.9 years, average recurrence age of 39.0 ± 15.4 years, and a median recurrence time of 6.0 (4.0, 9.0) years after resection of the primary tumor (**Figure 1**). Twenty-one percent of patients (20/96) exhibited recurrence between 10 to 20 years after the initial surgery, and one patient exhibited recurrence 23 years after the initial surgery. Among recurrent patients, 47% (45/96) had multiple nodules in recurrent site, and 58% (56/96) had metastases, of which 71% (40/56) were synchronous metastases (recurrence accompanied with metastases) and 34% (19/56) were implantation metastases. At the current follow-up, seven recurrent patients had died. The 112 non-recurrent patients included 75 PHEO patients, 33 PGL patients and 4 patients with both PHEO and PGL. Non-recurrent patients had an average diagnosis age of 40.5 ± 12.9 years and a median follow-up of 8.0 (7.0, 9.8) years. Germline mutations were screened in 182 patients by using next-generation sequencing (including genes *VHL, RET, NF1, SDHA, SDHB, SDHC, SDHD, SDHAF2, MAX, TMEM127, FH and KIF1B*) (8), and Sanger sequencing. 33% (60/182) patients carried pathogenetic mutations in genes including *SDHB, VHL, RET, MAX, SDHD* and *SDHA*.

### The Characteristics and Risk Factors of Recurrent PPGL

Compared with non-recurrent patients, recurrent patients exhibited at a younger diagnosis age (32.2 ± 14.9 in recurrent *vs*. 40.5 ± 12.9 in non-recurrent patients, p=0.000). Moreover, among the recurrent patients was a higher proportion of patients with primary tumor sizes ≥ 5cm (78% *vs*. 51% in non-recurrent patients, p=0.000), and a higher proportion of patients with gene mutations (42% *vs*. 26%, p=0.020), in particular in the *SDHB* gene (19% *vs*. 6% p=0.005). The proportion of patients with a primary tumor resection by laparotomy was higher than in the non-recurrent group (54% *vs*. 27% p=0.001) and the pathological features of the primary tumors of recurrent patients were more aggressive, manifesting more frequently as capsular invasion (20% *vs*. 5%, p=0.002), with vascular tumor emboli occurring more frequently (14% *vs*. 4%, p=0.018). Moreover, both the average Ki-67 count [4.5% (2%, 8%) *vs*. 1% (<1%, 2%), P=0.001] or the proportion of patients with an average Ki-67 count ≥ 3% (43% *vs*. 17%, p=0.001) were higher in recurrent than in non-recurrent patients. In contrast, there were no statistically significant differences in tumor functionality, levels of serum neuron specific enolase, primary tumor sites, proportion of patients presenting with typical symptoms, or *RET/VHL/SDHD/SDHA/MAX* mutation frequencies between the two groups (**Table 1**).

**Table 1:** Differences in clinical characteristics between pheochromocytoma/paraganglioma patients with and without recurrence.

Patients	Total patients	Recurrence group	No recurrence group	P value
Male	42% (87/208)	39% (37/96)	45% (50/112)	0.400
Age at diagnosis,y	36 ± 15	32.2 ± 14.9	40.5 ± 12.9	**0.000**
Follow-up time,y	7.0 (4.0,9.3)	6.0 (4.0,9.0)	8.0 (7.0,9.8)	
BPmax, mmHg				
Systolic BP	189 ± 39	196 ± 36	179 ± 51	0.203
Diastolic BP	115 ± 26	120 ± 25	108 ± 32	0.104
Typical symptoms^a^	80% (167/208)	77% (74/96)	72% (81/106)	0.911
Functionality				
NE, ug/24h.	166 (58,386)	220 (73,555)	127 (42,323)	0.099
E, ug/24h.	3.7 (2.7,7.6)	3.8 (2.5,7.4)	3.6 (2.7,10.4)	0.592
DA, ug/24h.	221 (156,319)	252 (166,327)	212 (149,304)	0.244
NSE, ng/mL	14.1 (11.0, 17.2)	14.7 (12.0,18.1)	12.7 (10.6,16.7)	0.801
Primary tumor size	5.8 ± 2.4	6.2 ± 2.3	5.6 ± 2.5	0.078
Tumor size≥5cm	64% (124/195)	78% (69/88)	51% (55/107)	**0.000**
Tumor site				
Left adrenal gland	28% (59/208)	30% (29/96)	27% (30/112)	0.585
Right adrenal gland	28% (60/208)	29% (27/96)	29% (33/112)	0.832
Bilateral adrenal glands	11% (23/208)	10% (10/96)	12% (13/112)	0.785
Paragangliomas	32% (66/208)	30% (29/96)	33% (37/112)	0.662
Multiple primary tumors	15% (31/208)	16% (15/96)	14% (16/112)	0.787
Laparotomy	36% (70/196)	54% (42/87)	27% (28/109)	**0.001**
Gene mutation	33% (60/182)	42% (33/78)	26% (27/104)	**0.020**
*SDHB*	12% (21/182)	19% (15/78)	6% (6/104)	**0.005**
*VHL*	6% (11/182)	9% (7/78)	4% (4/104)	0.210
*RET*	10% (19/182)	8% (6/78)	13% (13/104)	0.336
*SDHD*	3% (5/182)	3% (2/78)	3% (3/104)	0.896
*SDHA*	1% (2/182)	1% (1/78)	1% (1/104)	0.837
*MAX*	1% (2/182)	3% (2/78)	0% (0/104)	0.101
Capsular invasion	11% (21/186)	20% (15/76)	5% (6/110)	**0.002**
Vascular tumor embolus	9% (16/186)	14% (11/76)	4% (5/110)	**0.018**
Ki-67 count	1% (<1%, 3%))	4.5% (2%, 8%)	1% (<1%,2%)	**0.001**
Ki-67 count ≥3%	26% (40/154)	43% (23/54)	17% (17/100)	**0.001**

BP, blood pressure; a, the classic triad of headache, palpitation, and profuse sweating; NE, 24-hour urinary norepinephrine excretion (normal range: 17-41 μg/24 h); E, 24-hour urinary epinephrine excretion (normal range: 1.74-6.42 μg/24 h; DA, 24-hour urinary dopamine excretion (normal range: 121-331 μg/24 h); NSE, neuron specific enolase (normal range: 0-16.3 ng/mL). Bold values indicate significant P values.

**Figure 2:**
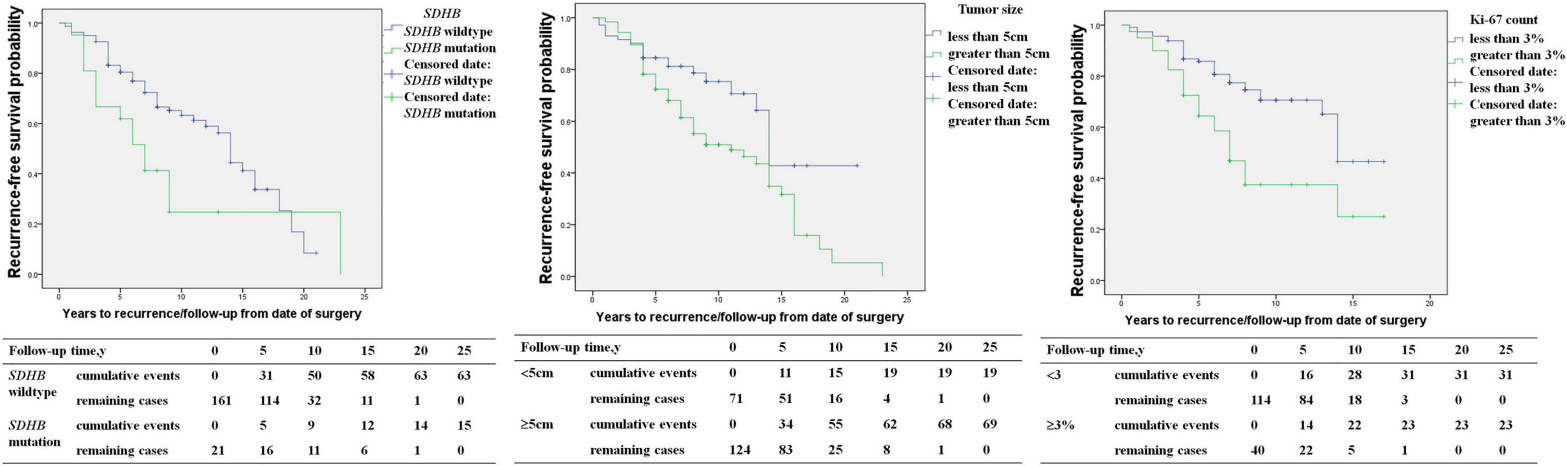
The recurrence-free survival in pheochromocytoma/paraganglioma patients (SDHB mutation patients *versus SDHB* wildtype patients; primary tumor size <5 *versus* primary tumor size ≥5cm; average Ki-67 count <3% *versus* Ki-67 count ≥3%).

*SDHB* mutation [HR 2.1 (1.2, 3.4), p=0.014], primary tumor size ≥5cm [HR 2.0 (1.2, 3.4), p=0.007], capsular invasion of the primary tumor [HR 2.0 (1.1, 3.7), p=0.016], and an average Ki-67 count ≥3% [HR 2.6 (1.5, 4.4), p=0.001] in the primary pathology were risk factors for recurrence in PPGL patients in univariable COX regression analysis (**Table 2**). *SDHB* mutation [HR 4.1 (1.7, 9.5), p=0.001), a primary tumor size ≥5cm [HR 2.3 (1.1, 4.7), p=0.028], and average Ki-67 count ≥3% [HR 2.6 (1.4, 4.9), p=0.003] represented independent predictors for recurrence in multivariate analysis (**Table 2** and **Figure 2**).

**Table 2:** Hazard ratios for the risk of recurrence of pheochromocytoma/paraganglioma.

Factors	Univariable			Multivariate		
	HR	95%CI	*P*	HR	95%CI	*P*
*SDHB* mutation	2.1	1.2, 3.4	**0.014**	4.1	1.7, 9.5	**0.001**
Tumor size≥5cm	2.0	1.2, 3.4	**0.007**	2.3	1.1, 4.7	**0.028**
Capsular invasion	2.0	1.1, 3.7	**0.016**	1.4	0.6, 3.3	0.424
Ki-67 count ≥3%	2.6	1.5, 4.4	**0.001**	2.6	1.4, 4.9	**0.003**

Bold values indicate significant P values.

### The Characteristics and Risk Factors of Recurrent PHEO

**Figure 3:**
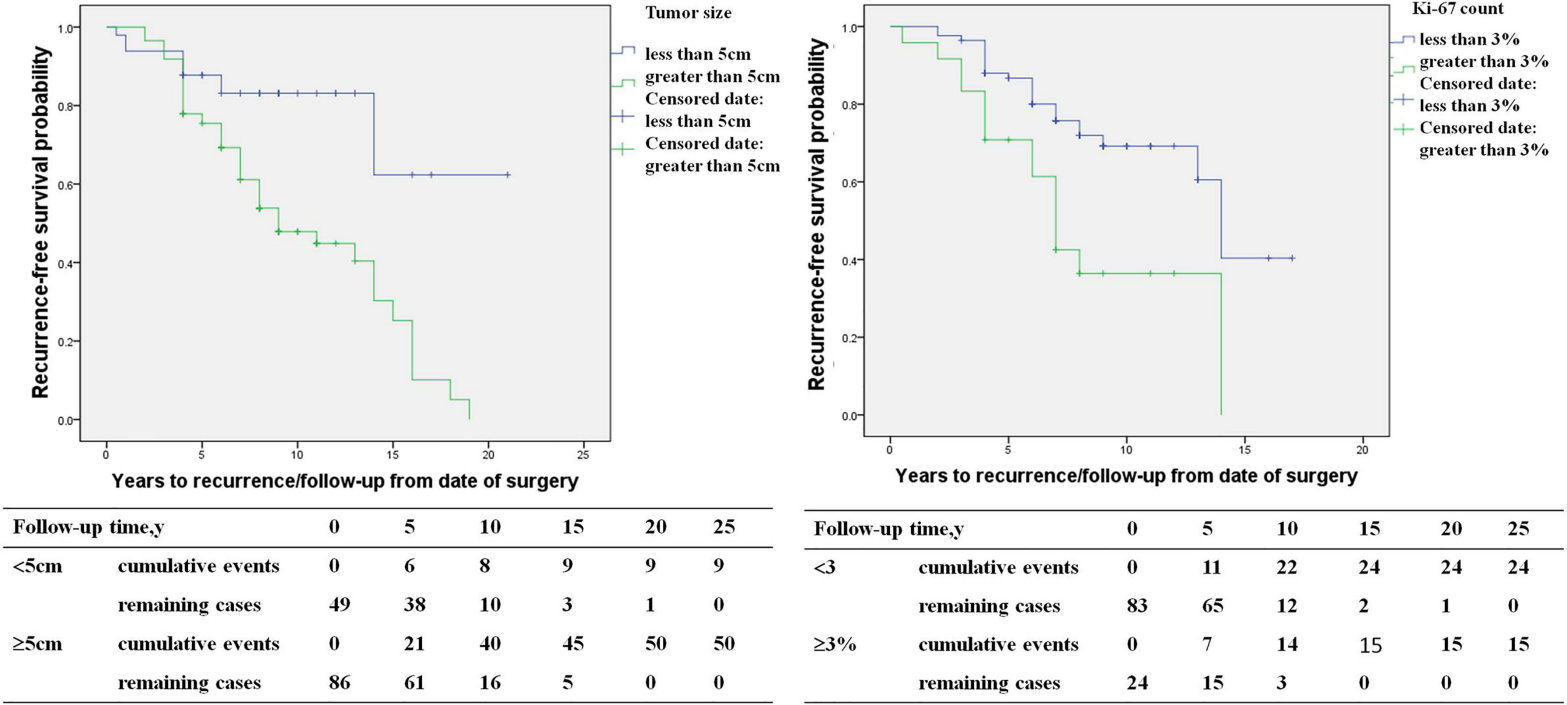
The recurrence-free survival (primary tumor size <5 *versus* primary tumor size ≥5cm, average Ki-67 count <3% *versus* Ki-67 count ≥3%) in pheochromocytoma patients.

The study included 146 PHEO patients, including 67 recurrent patients and 79 non-recurrent patients. Compared with non-recurrent PHEO patients, recurrent patients of PHEO presented at a younger diagnosis age (32.8 ± 15.3 in recurrent *vs*. 39.4 ± 13.3 in non-recurrent patients, p=0.007) and exhibited a larger primary tumor size (6.3 ± 2.3 *vs*. 5.5 ± 2.5 p=0.048). Among patients with recurrence there was a larger proportion of patients with a primary tumor size ≥5cm (75% *vs*. 46%). Both the average Ki-67 count [2% (1%, 5%) *vs*. 1% (1%, 2%), P=0.005] or the proportion of patients with an average Ki-67 count ≥3% (38% *vs*. 13%, p=0.002) were higher in recurrent patients (**Table 3**). Primary tumor sizes ≥5cm [HR 5.1 (1.7,15.3), p=0.003] and an average Ki-67 count ≥3% of primary tumor [HR 2.4 (1.1, 5.2), p=0.035] were independent predictors for recurrence of PHEO in multivariate COX regression analysis (**Figure 3**).

**Table 3:** Differences in clinical characteristics between pheochromocytoma patients with and without recurrence.

Patients	Total patients	Recurrence group	No recurrence group	P value
Male	43% (63/146)	39% (26/67)	47% (37/79)	0.329
Age at diagnosis, y	36 ± 15.0	32.8 ± 15.3	39.4 ± 13.3	**0.007**
Follow-up time, y	7.0 (5.0,9.0)	6.0 (4.0,9.0)	8.0 (7.0,9.0)	**0.000**
Primary tumor size	5.8 ± 2.4	6.3 ± 2.3	5.5 ± 2.5	**0.048**
Tumor size≥5cm	64% (86/135)	75% (50/67)	46% (36/79)	**0.000**
Gene mutation	33% (42/126)	39% (21/53)	29% (21/73)	0.067
*SDHB*	6% (8/126)	9% (5/53)	4% (3/73)	0.226
*VHL*	8% (10/126)	13% (7/53)	4% (3/73)	0.062
*RET*	13% (19/126)	11% (6/53)	18% (13/73)	0.315
*MAX*	2% (2/126)	4% (2/53)	0% (0/73)	0.090
Tumor site				
Left adrenal gland	41% (59/146)	45% (29/67)	38% (30/79)	0.515
Right adrenal gland	40% (60/146)	40% (27/67)	41% (32/79)	0.857
Bilateral adrenal glands	16% (23/146)	15% (10/67)	16% (13/79)	0.800
Multiple primary tumors	18% (27/146)	16% (11/67)	20% (16/79)	0.552
Laparotomy	25% (34/136)	40% (24/60)	13% (10/76)	**0.000**
Capsular invasion	9% (12/130)	15% (8/52)	5% (4/78)	0.064
Vascular tumor embolus	8% (11/130)	12% (6/52)	6% (5/78)	0.303
Ki-67 count	1% (1%,2%))	2% (1%, 5%)	1% (1%,2%)	**0.005**
Ki-67 count ≥3%	22% (24/108)	38% (15/39)	13% (9/69)	**0.002**

Bold values indicate significant P values.

### The Characteristics and Risk Factors of Recurrent PGL

**Figure 4:**
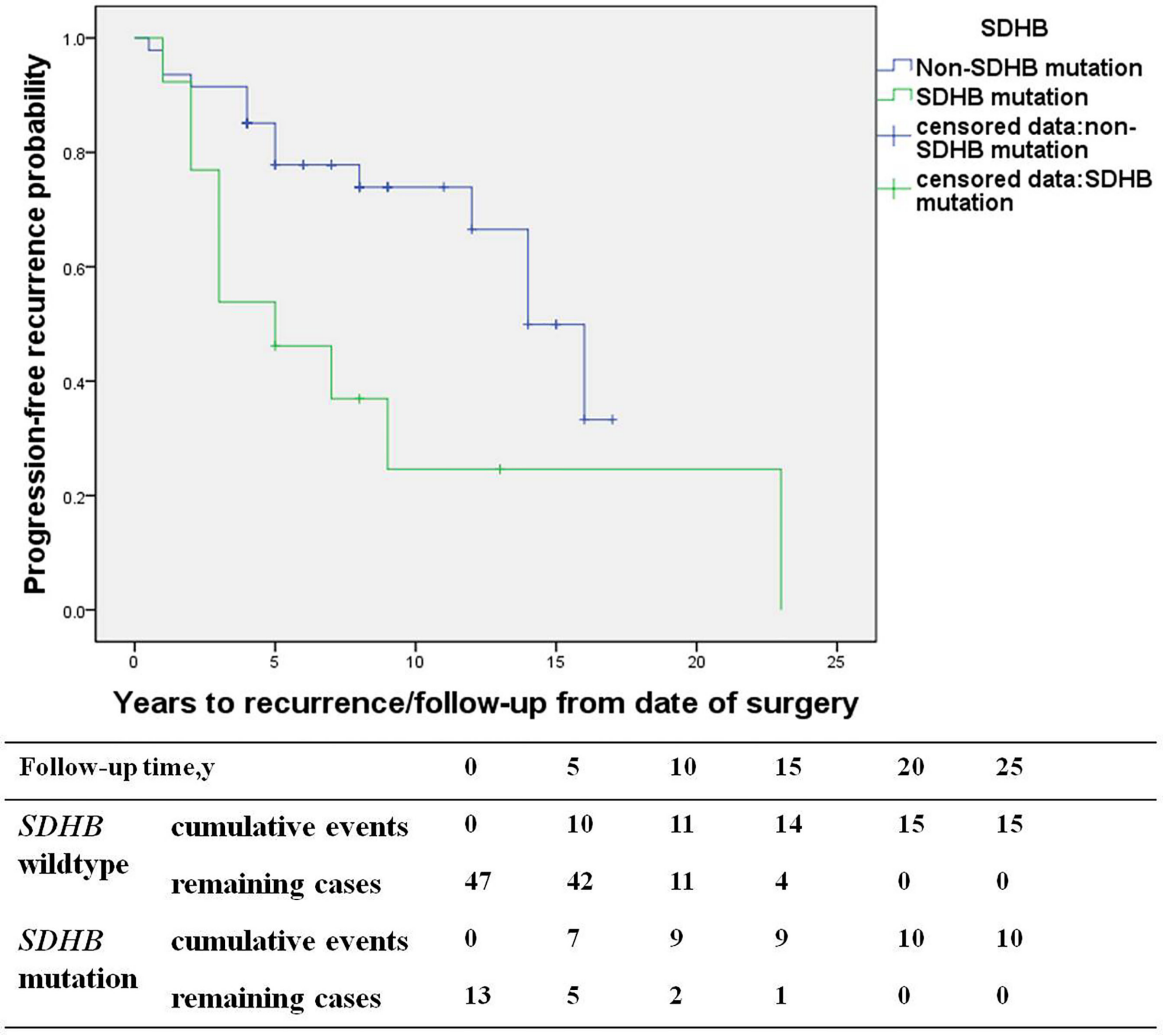
The recurrence-free survival (*SDHB* mutation patients *versus SDHB* wildtype patients) in paraganglioma patients.

The study consisted of 66 PGL patients, including 29 recurrent patients (27 retroperitoneal PGL, 1 bladder PGL, and 1 cardiac PGL) and 37 non-recurrent patients (33 retroperitoneal PGL, 3 bladder PGL, and 1 cardiac PGL). Compared with the non-recurrent PGL patients, recurrent patients of PGL presented at a younger diagnosis age (30.7 ± 13.8 in recurrent patients *vs*. 43.7 ± 11.9 years in non-recurrent patients, p=0.000) and a higher proportion of patients with a *SDHB* mutation (40% *vs*. 9%, p=0.004). The primary tumors pathological characteristics of recurrent patients were more aggressive, manifesting more frequently as capsular invasion (29% *vs*. 6% in non-recurrent patients, p=0.023), vascular tumor emboli happened more frequently (17% *vs*. 0%, p=0.008), and both the average Ki-67 count [3% (1%, 4%) *vs*. 1% (1%, 3%), p=0.021] or the proportion of patients with an average Ki-67 count ≥ 3% was higher in recurrent patients (53% *vs*. 23%, p=0.049) (**Table 4**). *SDHB* mutations [HR 4.6 (1.5, 13.9), p=0.007] were independent predictors for PGL recurrence in multivariate analysis (**Figure 4**).

**Table 4:** Clinical characteristics of paraganglioma with and without recurrence.

Patients	Total patients	Recurrence group	No recurrence group	P value
Male	39% (26/66)	38% (11/29)	19% (15/37)	0.830
Age at diagnosis, y	37 ± 14	30.7 ± 13.8	43.7 ± 11.9	**0.000**
Follow-up time, y	7.0 (4.0,9.3)	5 (2.5,10.0)	7.0 (5.5,9.5)	
Primary tumor size	5.8 ± 2.3	5.8 ± 2.4	5.6 ± 2.3	0.654
Tumor size≥5cm	61% (39/64)	66% (19/29)	57% (20/35)	0.494
Gene mutation	30% (18/60)	44% (11/25)	23% (8/35)	0.082
*SDHB*	22% (13/60)	40% (10/25)	9% (3/35)	**0.004**
Multiple primary tumors	12% (8/66)	14% (4/29)	11% (4/37)	0.713
Laparotomy	55% (35/64)	67% (18/27)	46% (17/37)	0.100
Capsular invasion	15% (9/60)	29% (7/24)	6% (2/36)	**0.023**
Vascular tumor embolus	8% (5/60)	17% (5/29)	0% (0/36)	**0.008**
Ki-67 count	2% (1%, 3%)	3% (1%, 4%)	1% (1%, 3%)	**0.021**
Ki-67 count ≥3%	32% (16/50)	53% (8/15)	23% (8/35)	**0.049**

Bold values indicate significant P values.

### The Characteristics of Metastatic Patients

**Figure 5:**
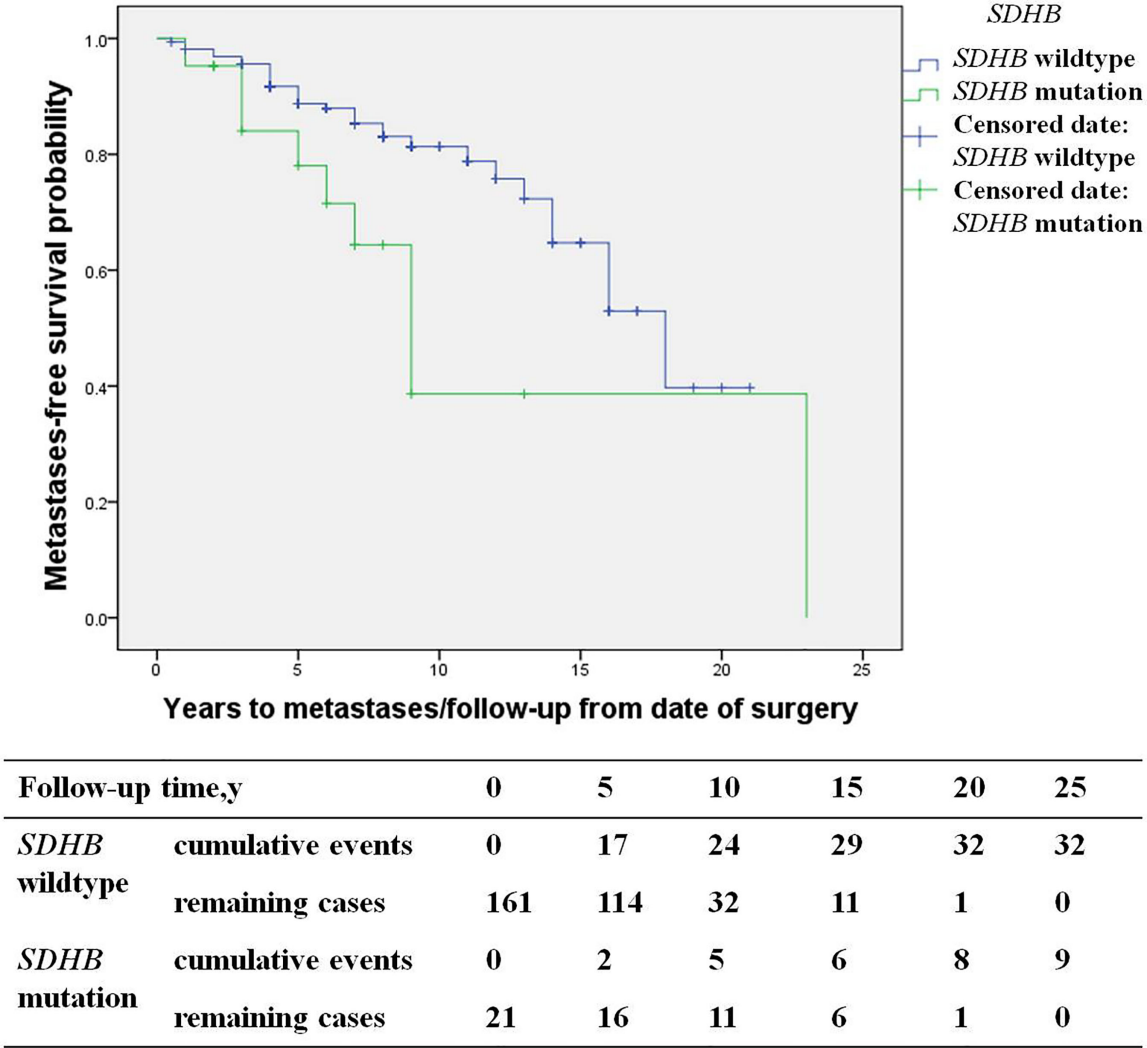
Progression-free metastases (*SDHB* mutation patients *versus SDHB* wildtype patients) in PPGL patients.

Among recurrent patients, 58% (56/96) had metastases. Most of these patients exhibited multi-system metastases, including 34% (19/56) implantation metastases, 34% (19/56) lung metastases, 30% (17/56) bone metastases, 27% (15/56) lymph node metastases, and 21% (12/56) liver metastases. *SDHB* mutations [HR 3.6 (1.3, 10.2), p=0.017] were an independent predictor for metastases by multivariate analysis (**Figure 5**). The study included 19 patients with implantation metastases. These patients exhibited an average diagnosis age of 37.1 ± 15.3 years, an average recurrence age of 42.8 ± 15.5 years, and a median recurrence time of 5 (4, 7) years. The average primary tumor diameter was 6.1 ± 1.8 cm, and 89% (17/19) were PHEO, of which 37% (7/19) were left adrenal PHEO, 42% (8/19) were right adrenal PHEO, 10% (2/19) were bilateral adrenal PHEO. Ten percent of patient with implantation metastases (2/19) had distant metastases, with one patient exhibiting a liver metastasis and the other exhibiting bone and lymph node metastasis. No independent predictors for implantation metastases were found in multivariate analysis.

### Treatment of Recurrence

**Figure 6:**
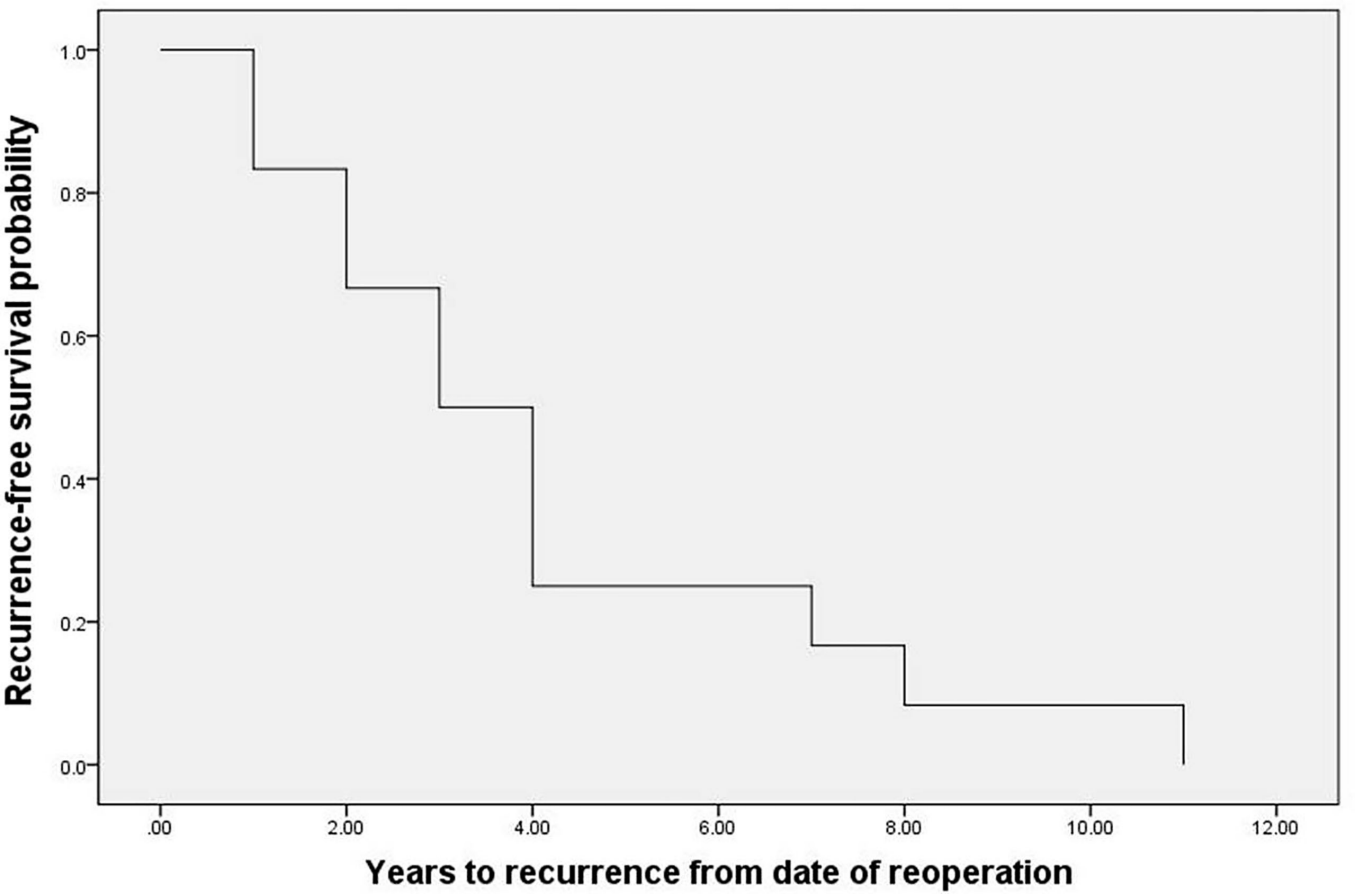
The recurrence-free survival probability after reoperations.

Thirty-two percent of recurrent patients (31/96) underwent surgery, of which 81% (25/31) patients underwent surgery only. However, 48% (12/25) exhibited recurrence after reoperation with a median time of recurrence of 3 (1.8, 4.2) years (**Figure 6**). Recurrent lesions in 19% (6/25) of these recurrent patients had not been completely resected, and patients therefore received MIBG, radiotherapy, and chemotherapy, amongst other therapeutic options. Forty-three percent of recurrent patients (41/96) could not undergo surgery. Of these patients, 78% (32/41) patients received MIBG treatment, 12% (5/41) patients underwent chemotherapy/targeted therapy, and 10% (4/41) patients underwent radiotherapy. Fourteen percent of recurrent patients (13/96) were recommended for surgery, but refused and did not receive any other treatment. Of these patients, five developed metastases during the observation period, one patient could not undergo surgery during the observation peried as the lesion progressed and invaded surrounding organs, and 2 patients died of metastases. Compared with the treated patients, the risk of metastases and death was significantly increased in the untreated patients (42% *vs*. 25%, p=0.030, and 15% *vs*. 8%, p=0.003, respectively). The treatment records of the remaining 11% (11/96) recurrent patients were unclear due to lost to follow-up after the diagnosis of recurrence.

## Discussion

Recurrence of PPGL after resection occurred in 13.3% patients who were followed up for more than five years after tumor resection in our center (unpublished data). A recent systematic review showed that the overall rate of recurrent PPGL was 0.98 events/100 person-years, which suggested the risk of recurrence after complete resection was previously underestimated (9). In a meta-analysis of 13 studies, the mean time to recurrence was approximately 4 years (0.5-12) (6). In our study, recurrent patients exhibited a median recurrence time of 6.0 (4.0, 9.0) years after resection of the primary tumor, which is longer than previous reports (2, 6). Our data suggest that the follow-up of all patients after tumor resection should be at least 10-20 years, as recurrences may occur after a prolonged time after initial treatment.

In our study, we found *SDHB* mutation, primary tumor sizes ≥5cm, and average Ki-67 counts ≥3% in the primary tumor were independent predictors of recurrence. The results in our study were similar to previous reports (3, 7, 10–13). Moreover, we separately studied the predictors of PHEO and PGL, and found that the risk factors for recurrence of PHEO and PGL were different. The risk of recurrence was 5.1- and 2.4-fold higher in PHEO patients with primary tumor sizes≥ 5cm and average Ki-67 counts ≥3%, respectively. *SDHB* mutations were predictors for recurrence in PGL patients and not for PHEO, with the risk of recurrence being 4.6 fold higher in PGL patients with *SDHB* mutation than those without. Recent studies have shown that higher Pheochromocytoma of the Adrenal Gland Scaled Score (PASS) and Grading system for Adrenal Pheochromocytoma and Paraganglioma(GAPP) score were significantly associated with recurrence, and it was, respectively, 1.2- and 3.4-fold higher in patients with higher PASS and GAPP scores (13, 14).

In our study, 19 patients unfortunately exhibited implantation metastases which are considered to be related to tumor cells scattered in peritoneal cavity during surgical resection (15). The initial surgery of most patients was not performed in our hospital. Laparoscopic surgery may be more challenging if the tumor is large since these tumors more likely to be broken and tumor cells scattered when manipulated. The guideline recommends open resection for large (>6 cm) PHEO and PGL to ensure complete tumor resection, preventing tumor rupture and avoiding local recurrence (16). However, several studies have suggested that larger PPGL may be amenable to laparoscopy with safety levels similar to those for smaller tumors (4, 17). Here, we did not find that laparoscopic surgery can increase the risk of recurrence. The key to preventing implantation metastases is an endocrine surgeon with expertise in laparoscopic techniques and operating on PPGL. Furthermore, in cases of tumor rupture during surgical resection, careful follow-up is mandatory.

Recurrence is difficult to treat especially if diagnosis is delayed. Unfortunately, surgery was not an option in 43% of recurrent patients included in this study. Several reasons can lead to difficulty of recurrence treatment, for instance, 47% recurrent patients had multiple nodules in recurrent site in our study, recurrent patients were often accompanied by metastases, 58% had metastases in our study (7). Although treatment of recurrences is challenging, patients should be treated as soon as possible after detection of recurrence as we found that treatment significantly decreased the risk of metastases and death compared to untreated patients.

There were some limitations in our study. Firstly, local-regional recurrence referred to a reappearance of disease at the original site after complete surgical resection which had been confirmed by negative biochemical and imaging tests. However we cannot completely exclude the situation that a separate, metachronous PPGL arising in the same location. Another weakness of our study was the lack of PASS/GAPP scores which may be beneficial to predicting tumor recurrences.

## Conclusion

Long-term follow-up in all PPGL patients is necessary. PHEO patients with primary tumor sizes ≥5cm, average Ki-67 counts ≥3%, and PGL patients with *SDHB* mutation exhibited an elevated risk of recurrence. Recurrence is difficult to treat as it is often accompanied by multiple nodes in recurrent sites, distant metastases, and implantation metastases, however it is recommended as it can reduce the risk of further metastases and death.

## Data Availability Statement

The original contributions presented in the study are included in the article/supplementary material. Further inquiries can be directed to the corresponding author.

## Ethics Statement

The study was approved by the Institutional Review Board of Peking Union Medical College Hospital(S-K431). Written informed consent was obtained from the patients included.

## Author Contributions

YC performed the study and drafted the manuscript. AT contributed to the concept and design for the study. YG, XM, SC, and LL contributed to the manuscript preparation. XC prepared histopathological results. All authors contributed to the article and approved the submitted version.

## Funding

This work was supported by CAMS Innovation Fund for Medical Sciences (CIFMS) of China (Grant No. 2021-I2M-C&T-B-002, and 2017-I2M-1-001), and National Natural Science Foundation of China (Grant No. 81770427, and 82070822).

## Conflict of Interest

The authors declare that the research was conducted in the absence of any commercial or financial relationships that could be construed as a potential conflict of interest.

## Publisher’s Note

All claims expressed in this article are solely those of the authors and do not necessarily represent those of their affiliated organizations, or those of the publisher, the editors and the reviewers. Any product that may be evaluated in this article, or claim that may be made by its manufacturer, is not guaranteed or endorsed by the publisher.
